# High-Altitude Pulmonary Edema in Two Pediatric Patients with Pre-Existing Lung Disease

**DOI:** 10.3390/pediatric16020023

**Published:** 2024-04-05

**Authors:** Ali Alsuheel Asseri, Marei Assiri, Norah Alshehri, Noha Saad Alyazidi, Ahmed Alasmari, Saud Q. Alshabab, Nada Abdullah Asiri

**Affiliations:** 1 Department of Child Health, College of Medicine, King Khalid University, Abha 62529, Saudi Arabia; 2 Departments of Pediatrics, Abha Maternity and Children Hospital, Abha 62562, Saudi Arabia; abummm99100@gmail.com (M.A.); noura.allshehri@gmail.com (N.A.); nalyazidi@moh.gov.sa (N.S.A.); ahmedalasmari18@gmail.com (A.A.); 3 College of Medicine, King Khalid University, Abha 62529, Saudi Arabia; dr.saud59@gmail.com (S.Q.A.); nadaabdullahasiri8@gmail.com (N.A.A.)

**Keywords:** high-altitude pulmonary edema, children, chronic lung disease, Saudi Arabia, Abha

## Abstract

Background: The illnesses associated with changes in barometric pressure can be classified into three types: acute mountain sickness, high-altitude pulmonary edema (HAPE), and high-altitude cerebral edema. HAPE is a rare form of pulmonary edema that occurs in susceptible individuals after arriving at altitudes over 2500 m above sea level (m). Only a few studies have reported classical HAPE among children with underlying cardiopulmonary comorbidities. In this study, we report two pediatric cases of classical HAPE that occurred immediately upon arriving at Abha city (with an average elevation of 2270 m above sea level). Notably, both patients possessed underlying chronic lung diseases, raising crucial questions about susceptibility factors and the early onset manifestations of HAPE. Case: Two pediatric cases of HAPE are presented. The first patient, with a medical history of repaired right congenital diaphragmatic hernia and subsequent right lung hypoplasia, developed HAPE following their ascent to a high altitude. The second patient, diagnosed with diffuse lung disease of unknown etiology, experienced HAPE after a rapid high-altitude ascent. Both patients resided in low-altitude areas prior to ascent. The initial emergency room assessment revealed that both patients had severe hypoxia with respiratory distress that mandated the initiation of respiratory support and 100% oxygen therapy. They required intensive care unit admission, improved after 5 days of hospitalization, and were sent home in good condition. Conclusion: HAPE is a complex, potentially life-threatening high-altitude illness with diverse clinical presentations and variable risk factors. This case report sheds light on a potential predisposition factor—pre-existing lung disease—in children experiencing severe HAPE. While further validation is crucial, this valuable insight opens doors for improved preventative strategies and informed medical decisions for children with pre-existing lung conditions traveling to high altitudes.

## 1. Introduction

Abha city is the capital of the Aseer region of the Kingdom of Saudi Arabia. It is located at an average elevation of 2270 m (7500 feet) above sea level and is among the highest cities in the country. Traveling to high altitudes presents a unique physiological challenge. As one ascends, barometric pressure progressively decreases, leading to a corresponding reduction in the partial pressure of oxygen in inspired air (PO_2_) [1,2,3]. This decreased oxygen availability can pose potential health risks—namely, high-altitude illnesses—especially to children with underlying cardiopulmonary diseases [4]. The illnesses associated with changes in barometric pressure can be classified into three types: acute mountain sickness, high-altitude pulmonary edema (HAPE), and high-altitude cerebral edema [5]. HAPE is a rare form of pulmonary edema that occurs in susceptible individuals after arriving at altitudes over 2500 m above sea level (m). The exact incidence of HAPE among children is unknown; however, it varies with individual susceptibility, ascent rate, altitude, and the presence of cardiopulmonary disorders [1,2,5,6,7]. There are few reported cases in the literature of children developing HAPE after ascending to a moderate altitude (<2400 m; 7870 feet) [8].

Generally, three distinct types of HAPE have been proposed: classic HAPE from rapid ascent, re-entry HAPE upon returning to high altitude, and high-altitude resident pulmonary edema (HARPE) triggered by respiratory illness at high altitude, even without changing elevation [7,9]. Only a few studies have reported classical HAPE among children with underlying cardiopulmonary comorbidities [4]. In this study, we report two pediatric cases of classical HAPE that occurred immediately upon arriving at Abha city. Notably, both patients possessed underlying chronic lung diseases, raising crucial questions about susceptibility factors and early onset manifestations of HAPE.

## 2. Cases

**Figure 1:**
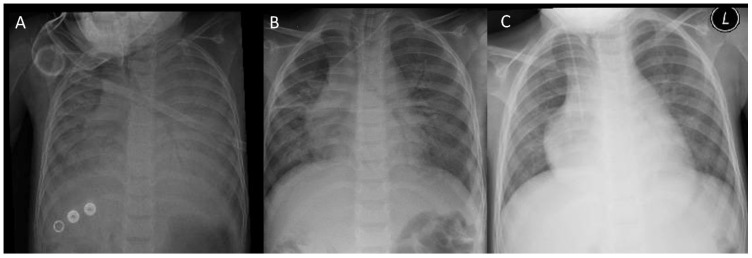
Patient 1: (**A**) frontal CXR showing bilateral opacities silhouetting bilateral hemidiaphragm and cardiac borders; (**B**) follow-up CXR after 2 days showing significant improvement with minimal residual patchy infiltration involving both lungs; (**C**) CXR on day 5 of admission showing complete resolution of the lung opacities. L = left, CXR = chest X-ray.

Case 1: A 3-year-old female child presented to the emergency room at Abha Maternity and Children Hospital with an acute onset of shortness of breath and cough for a 12 h duration after arriving at Abha city. Her past medical history revealed that the patient had a repaired right congenital diaphragmatic hernia (CDH) (repaired at the age of 6 months), recurrent pneumonia, and recurrent viral-induced wheezy chest. Prior to the diagnosis of CDH, she had experienced recurrent episodes of severe pneumonia that required pediatric intensive care unit admission and intubation twice. Chest imaging at that time revealed right lung hypoplasia with elevated right hemidiaphragm, and she was referred to the pediatric surgery team. The CDH diagnosis was established, and she underwent surgical repair with prolonged PICU admission for 2 months and then went home off oxygen. Since then, she has had attacks of viral-induced wheezy episodes that needed emergency room visits, but has never been admitted. She lives at a low altitude (70 m above sea level). The patient is fully immunized. During physical examination, she was in acute distress and had severe hypoxia with an SpO_2_ of 40% breathing room air; furthermore, her heart rate was 190 beats per minute and blood pressure was 100/62 mmHg (normal for age). Her weight was 11 kg, in the 10th percentile for her age. Her lung exam revealed bilateral diminished air entry with diffuse inspiratory crackles. Chest X-ray (CXR) on admission (Figure 1A) showed bilateral patchy opacities.

Detailed laboratory data at the time of admission are shown in Table 1. Due to persistent hypoxemia and severe respiratory distress, the patient was intubated and mechanically ventilated. The differential diagnosis at admission included acute respiratory distress syndrome, pneumonia, cardiogenic pulmonary edema, and noncardiogenic edema. The patient was started on broad-spectrum antibiotics, IV methylprednisolone, and nebulization therapy. Echocardiography showed normal heart function and structure without pulmonary hypertension, and this was performed on day 2 of admission. On the second day, her oxygenation and chest opacities improved. The repeated CXR after 6 h (Figure 1B) revealed great improvement compared with the initial CXR. A trial of extubation was performed, which the patient tolerated, and the patient was removed from oxygen on day 4 of admission. Given the history of an ascent to high altitude, a rapid improvement of the clinical condition, and radiological findings, a diagnosis of HAPE was proposed. All infectious workups returned normal, as did the echocardiographic study. On the fifth day, the patient was discharged to their home on room air with a normal heart rate, with their CXR showing a significant improvement of patchy opacities (Figure 1C). Informed consent was obtained from the patient and patient’s parents.

**Figure 2:**
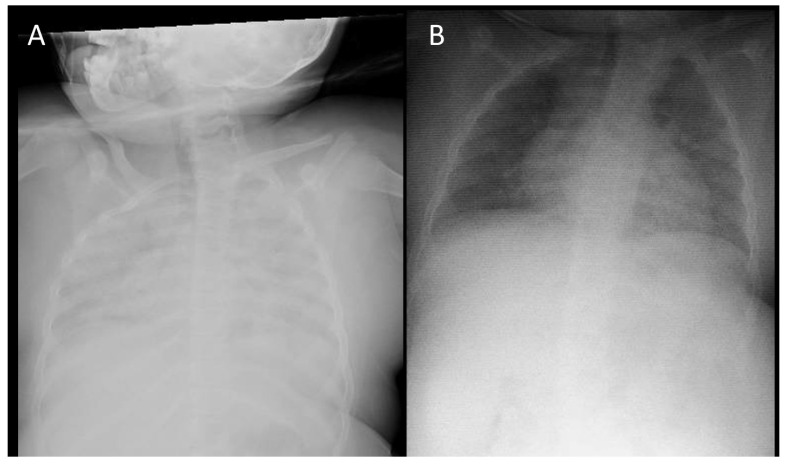
Patient 2: (**A**) frontal CXR showing severe bilateral air space opacities; (**B**) follow-up CXR after 4 days showing significant improvement.

Case 2: A 10-year-old female presented with a history of progressive dyspnea and productive cough 6 h after arriving at Abha city (2270 m above sea level). The family lives in a low-altitude area (525 m above sea level) and the patient reported a history of fever, cough, and mild shortness of breath. Her past medical history revealed that the patient had recurrent pneumonia since early infancy and had undergone a full investigation without any exact underlying pulmonary disease being identified. The immune workup was negative, as was the result of whole-exome sequencing. Previous advanced chest imaging (contrasted CT-chest) revealed diffuse air space opacities with thickened interstitium. Her vital signs on admission to the emergency room were a heart rate of 145 bpm, respiratory rate of 28 breaths/min, blood pressure of 110/65 mmHg, temperature of 36.5 °C, and SpO_2_ of 71% on RA. The lung examination revealed severe respiratory distress with crackles most commonly heard at the lung bases. Neurologically, the patient was fully awake with a Glasgow coma scale of 15/15. The laboratory tests are summarized in Table 1. Chest X-ray revealed bilateral extensive heterogeneous opacities that involved both lung fields (Figure 2A).

She was started on a high-flow nasal cannula (HFNC) at 15 L per minute with a 100% fraction of inspired oxygen due to persistent hypoxemia and severe respiratory distress, despite being on high-flow oxygen. The differential diagnosis included complicated viral pneumonia, asthma, and noncardiogenic edema. She was started on broad-spectrum antibiotics (ceftriaxone and vancomycin), as well as intravenous methylprednisolone.

A nasopharyngeal (NP) swab for SARS-CoV-2, respiratory syncytial virus, and influenza A and B PCRs was sent. On the second day of admission, the patient’s condition improved with successful weaning from HFNC, a partial resolution of respiratory symptoms, and an oxygen saturation of 97% on a 4 LPM simple face mask. On the second day of admission, cardiology was consulted to rule out structural heart defects and assess for the presence and severity of pulmonary hypertension. The patient underwent echocardiography that showed a normal cardiac structure and function without evidence of pulmonary hypertension or pulmonary artery anomalies. On the fourth day of admission, the NP viral panel PCR result returned negative, and the chest X-ray revealed a complete resolution of opacities (Figure 2B). Given the lack of cardiac defects as well as a negative respiratory viral panel, her history of travelling from low-altitude areas to high-altitude areas, and her rapid improvement with oxygen therapy, the patient was discharged home with a diagnosis of classical HAPE. Informed consent was obtained from the patient’s parents before writing this report.

## 3. Discussion

HAPE is a rare and potentially fatal form of non-cardiogenic pulmonary edema occurring in susceptible individuals exposed to hypobaric hypoxia. This triggers uneven pulmonary vasoconstriction and subsequent capillary damage due to increased pulmonary capillary permeability [1,3,5,10]. Three HAPE phenotypes have been proposed: classical, re-entry, and HARPE. Thus far, we have reported three pediatric patients who have developed re-entry HAPE and had an excellent recovery [9]. Additionally, we report two Saudi pediatric patients who developed classical HAPE and had pre-existing lung diseases in this study. To the best of our knowledge, this is the first report of classical HAPE in Saudi pediatric patients with pre-existing lung diseases.

Several risk factors of HAPE have been reported, including previous episodes of HAPE, recent upper respiratory tract infection, obesity, rapid ascent, obstructive sleep apnea, nocturnal hypoxemia, residence at an altitude below 900 m, and underlying cardiopulmonary diseases [6,7,11]. Our cases cast a new light on the presence of pre-existing lung disease as a risk factor for HAPE among children, which suggests that existing lung disease may be a previously underestimated risk factor for HAPE in children, with important implications for diagnosis and prevention.

With regard to clinical presentations, HAPE usually presents with cough, dyspnea, cyanosis, exercise intolerance, and crackles on physical examination, typically occurring within 2–5 days of ascent [6,12]. In HAPE cases, a chest radiograph will indicate findings of pulmonary edema. Notably, our patients experienced a noticeably faster onset of HAPE symptoms and exhibited a greater degree of severity than is typically observed. Additionally, the lengths of stay for our patients were 5 days longer when compared with the published case reports [8,9,13,14]. The presence of lung disease is the likely reason for this atypical presentation. However, more research is needed to validate these observations.

At high altitudes, multiple organ systems—such as the lungs, heart, kidneys, and hematological system—experience physiological adaptations in response to hypobaric hypoxia. The lungs are particularly crucial in the early stages of acclimatization to this hypoxic environment [6]. These immediate changes include a hypoxic ventilatory response, hypoxic pulmonary vasoconstriction (HPV), and changes in the oxygen affinity of hemoglobin [3,6]. Uneven HPV causes increased pulmonary capillary permeability in capillaries that are not protected from high pulmonary artery pressure and, subsequently, capillary failure with the leakage of plasma and red blood cells into the alveoli [3]. This type of over-perfusion edema occurs in lung regions with diminished HPV and, potentially, a weaker intrinsic vascular tone. While a comprehensive understanding of the molecular mechanisms driving these changes remains elusive, two potential hypotheses have been proposed. These hypotheses center on the interplay between increased endothelin-1 and reactive oxygen species (ROS) production, alongside a decrease in nitric oxide (NO) bioavailability [15,16,17]. The NO causes vasodilation while ROS and endolthelin-1 constrict pulmonary vasculatures, and the alteration of these molecules due to hypoxia causes uneven HPV and, subsequently, capillary leaks [15,16,17,18]. It is more prevalent in patients with congenital right pulmonary artery agenesis, unilateral pulmonary artery hypoplasia, or restricted pulmonary vasculature due to scoliosis or chest wall deformities [10]. The presence of HAPE in our patients occurred within hours of arriving at high altitude and was severe. It is possible, therefore, that the presence of underlying lung diseases played a significant role in the prompt onset and increased severity of HAPE in our patients. Case 1 had lung hypoplasia due to a right congenital diaphragmatic hernia (CDH), which contributed to the occurrence of HAPE. Notably, neither case showed evidence of pulmonary hypertension or underlying congenital heart disease. One interesting finding was that case 1 had HAPE with the left side being more involved than the right side, supporting the notion that high flow and pressure are important in the pathogenesis of HAPE. This is in accordance with earlier observations, which showed that an absent/hypoplastic right pulmonary artery is a risk factor for severe HAPE at even moderate altitudes [7].

While definitively proving that pre-existing lung disease is a risk factor of HAPE is challenging, our cases suggest a potential link between the impact of pre-existing lung disease on the pulmonary vasculature and the development of HAPE. Existing evidence from congenital diaphragmatic hernia-associated PHTN points towards structural changes such as a reduced artery density and thickened walls [19], which could contribute to this vulnerability in case 1. Moreover, a robust body of research has established a significant association between increased HAPE prevalence and left-to-right cardiac anomalies leading to pulmonary vascular hyper-perfusion, including atrial and ventricular septal defects, unilateral pulmonary artery absence, and patent ductus arteriosus [10,11,20]. Despite this correlation, further investigation is necessary to elucidate the links between specific cardiopulmonary disorders and the incidence of HAPE. Though our cases raise the possibility of vascular complications from pre-existing lung disease influencing patients’ susceptibility to HAPE, more research is needed to establish this connection and elucidate the specific mechanisms involved.

Due to the fatal outcome of HAPE if left untreated or misdiagnosed, more effort is needed to identify susceptible individuals; thus, relevant preventive measures should be implemented. Previous HAPE is the most important predictor of HAPE, and those who have had HAPE should follow the recommended preventive measures to avoid HAPE, including modest ascent rates of 275–300 m (900–1000 feet) per day, a rest day every 1000 m (3000 feet), and sleep at the lowest possible altitude [5,21]. Clinical trials have confirmed the effectiveness of acetazolamide, sildenafil, and nifedipine for both prophylactic and therapeutic interventions against HAPE in adults [5]. However, the data among children are scarce. A recently published review recommended treating the underlying predisposing conditions to HAPE, including addressing issues such as nocturnal hypoxemia at high altitude, sleep apnea, and pulmonary hypertension [7]. Additional research is needed to better understand the risk factors of HAPE among children and establish evidence-based therapeutic and preventive measures.

## 4. Conclusions

In conclusion, HAPE is a complex and potentially life-threatening high-altitude illness with diverse clinical presentations and variable risk factors. Recognizing its multifaceted nature and understanding the factors influencing individual susceptibility are crucial for optimizing preventative measures and ensuring safe high-altitude travel. This case report sheds light on a potential predisposition factor—pre-existing lung disease—in children experiencing severe HAPE. While further validation is crucial, this valuable insight opens doors for improved preventative strategies and informed medical decisions for children with pre-existing lung conditions traveling to high altitudes.

## Figures and Tables

**Table 1:** Laboratory data at the time of admission.

Variables	Reference Range	Patient 1	Patient 2
Blood and biochemical tests			
White blood cells (×10^9^/L)	4500–11,000	5.56	26.34
Absolute lymphocyte count per mm^3^	1000–4800	780	1120
Absolute neutrophil count per mm^3^	1800–7700	4040	24,920
Hemoglobin (g/dL)	12.0–16.0	10.8	11.4
Hematocrit	36–46%	38.0	36.3
Platelets (×10^9^/L)	150,000–450,000	189	358
ESR (mm/h)	0–13	4	80
BUN (mg/dL)	8.0–25	14	10
Creatinine (mg/dL)	0.30–1.00	0.36	0.39
Sodium (mEq/L)	135–145	137	136
ALT (IU/L)	10–55	12	9
AST (IU/L)	9.0–32	36	22
Albumin (g/dL)	3.4–5.4	3.49	3.6
pH	7.35–7.45	7.37	7.41
PCO_2_ (mmHg)	35–45	36.3	45
HCO_3_ (mEq/L)	22–26	21.1	27.4
Base excess (mEq/L)	−2–+2	−3.9	2.1
Oxygen saturation (%)	>95%	40	71
FiO_2_ (%)	-	100%	100%

ESR: erythrocyte sedimentation rate; BUN: blood urea nitrogen; ALT: alanine aminotransferase; AST: aspartate aminotransferase; pH: potential of hydrogen; PCO_2_: partial pressure of carbon dioxide; HCO_3_: bicarbonate; FiO_2_: fraction of inspired oxygen.

## Data Availability

The data that support the findings of this study are available from the corresponding author upon reasonable request.
